# Prognostic Value of Liver Biomarkers in Hepatocellular Carcinoma Patients Undergoing Yttrium 90 Transarterial Radioembolization (TARE): A Retrospective Pilot Study

**DOI:** 10.7759/cureus.61904

**Published:** 2024-06-07

**Authors:** Maria del Pilar Bayona Molano, Marcin Kolber, Juana V Barrera, Muhammad R Akram, Mhd Wisam Alnablsi, Tanya Pothini, Riad Salem, Amit G Singal

**Affiliations:** 1 Interventional Radiology, University of Texas Southwestern Medical Center, Dallas, USA; 2 Interventional Radiology, Midstate Radiology Associates, Meriden, USA; 3 Radiology, University of California San Francisco, San Francisco, CA, USA; 4 Radiology/Ophthalmology, University of Texas Southwestern Medical Center, Dallas, USA; 5 Radiology, Northwestern University, Chicago, USA; 6 Digestive and Liver Diseases, University of Texas Southwestern Medical Center, Dallas, USA

**Keywords:** tare, liver function markers, y90, transarterial radioembolization, liver fibrosis markers, systemic inflammation indexes, albi score, liver biomarkers

## Abstract

Introduction: Hepatocellular carcinoma (HCC) is a common cause of cancer-related death worldwide. The prognosis for HCC depends on the tumor stage, and curative therapies are more accessible in the early stages. However, effective treatments are available even in advanced stages. Transarterial radioembolization (TARE) is an alternative to transarterial chemoembolization (TACE) with reduced risk and extended disease progression time. Identifying prognostic indicators and treatment response biomarkers remains crucial. The purpose of this study was to assess the association between biomarkers related to fibrosis, liver function, and immune inflammation with tumor response to yttrium 90 transarterial radiotherapy (Y90 or TARE) in patients with HCC.

Methods: This study enrolled patients who underwent Y90 radiotherapy for bridging, downstaging, or palliative treatment after discussion in a multidisciplinary tumor board. Using the modified Response Evaluation Criteria in Solid Tumors (mRECIST), tumor response was classified into two groups: “responders” (complete and partial response) and “non-responders” (stable and progressive disease). Logistic regression analysis was used to evaluate the association between predictors, biomarkers such as aspartate aminotransferase (AST)-to-platelet ratio index (APRI), fibrosis-4 (FIB-4), albumin-bilirubin (ALBI) score, model for end-stage liver disease (MELD) score, MELD sodium, and the systemic immune-inflammatory indexes, at established cut-offs and tumor response.

Results: Of 35 patients, 22 (63%) were Whites and non-Hispanics, 32 (91%) were diagnosed with cirrhosis, and 14 (40%) of these had a viral etiology. According to mRECIST, 18 (51%) patients were classified as “responders.” In multivariable logistic regression analysis, biomarkers associated with tumor response were ALBI score ≤-2.8 (odds ratio (OR) 6.1, 95%CI 2.7-14.4) and the neutrophil-to-lymphocyte ratio (NLR) ≤ 1.92 (OR 5.1, 95%CI 0.8-11.9). Biomarkers had moderate accuracy in predicting tumor response (C-statistic 0.75).

Conclusion: The ALBI score is a reliable predictor of treatment response following TARE. The NLR index may offer further prognostic information, and both biomarkers can be used in combination; however, further research in larger sample sets is needed.

## Introduction

Hepatocellular carcinoma (HCC) is the fourth most common cause of cancer-related death worldwide, with rising mortality in the United States and Europe [1]. The prognosis for HCC is significantly influenced by the stage of the tumor. Curative therapies are selectively accessible for those diagnosed in the early stages, underscoring the advantages of implementing surveillance programs [2]. Notably, efficacious treatments are available to manage HCC even in its advanced stages.

Transarterial radioembolization (TARE), also known as selective internal radiation therapy (SIRT), delivers radiation therapy directly to liver tumors through the arterial blood supply. Tiny beads coated with the radioactive isotope yttrium-90 are injected, lodging in the tumor's blood vessels to deliver localized radiation while minimizing exposure to healthy tissue. Initially used for palliative care, TARE's role is expanding to include curative treatments. This loco-regional therapy aims to prolong survival by slowing the tumor progression or acting as a bridge to more definitive therapies [3].

TARE presents an alternative to transarterial chemoembolization (TACE), offering a lower risk of post-embolization syndrome and a significantly longer time for disease progression. Although overall survival (OS) rates are similar between TARE and TACE, not all patients respond to these treatments [3]. This highlights the need to identify prognostic indicators and biomarkers to better predict treatment responses and tailor therapies accordingly.

There have been several proposed treatment response biomarkers, ranging from markers of fibrosis to liver function to inflammation.

HCC is a disease that predominantly occurs in the context of advanced fibrosis if not cirrhosis. Hepatic fibrosis due to chronic liver disease is characterized by excessive accumulation of collagen and extracellular matrix (ECM) proteins [4]. During liver injury, hepatic stellate cells (HSCs) activate in contractile myofibroblasts, promoting portal hypertension and contributing to vascular distortion caused by fibrogenesis with connective tissue accumulation, amplified inflammation, and HCC [5]. Several noninvasive indirect methods that are inexpensive, readily available, and easy to calculate have been developed for the assessment of liver fibrosis, including the aspartate aminotransferase (AST)-to-platelet ratio index (APRI) and the fibrosis-4 (FIB-4) score. APRI along with the albumin-bilirubin (ALBI) score have been used to evaluate the association between postoperative recurrence and mortality in patients with HCC within the Milan criteria [6].

Even among those with cirrhosis, increased liver dysfunction may present a worse prognosis and decreased response to treatments. The ALBI score assesses liver function in patients with HCC, without a need for subjective variables, such as ascites and encephalopathy [7]; ALBI has been used as a prognostic factor in patients with resectable or locally advanced HCC, but there is little data among patients undergoing TARE [8]. Other prognostic scores in patients with cirrhosis and HCC include the Model for End-Stage Liver Disease (MELD) score, which is widely used to prioritize patients for liver transplantation (LT).

Finally, among patients with HCC, the risk of recurrence and metastasis is influenced by several factors, including immune and inflammatory cells, such as neutrophils, platelets, and lymphocytes that contribute to tumor cell invasion [9]. Platelets protect circulating tumor cells (CTCs) from natural killer cell activity [9,10], neutrophils promote the adhesion and seeding of CTCs in distant organs, and the activation of lymphocytes induces cytotoxic activity and tumor cell death [9]. Several systemic inflammation indexes have been proposed as prognostic factors in HCC, including the systemic immune-inflammatory index (SII), neutrophil-to-lymphocyte ratio (NLR), and platelet-to-lymphocyte ratio (PLR) [9,10,11]. NLR has been found to be an independent risk of tumor recurrence after curative hepatectomy [12] and as a risk factor in five-year OS in resectable HCC receiving TACE [13].

The objective of this study was to explore the potential impact of combining pretreatment biomarkers associated with liver fibrosis, liver function, immune inflammation, and tumor response following TARE in patients with HCC. The goal is to provide insights into the effectiveness of using these predictors in predicting tumor response and optimizing the treatment outcome for patients diagnosed with HCC.

## Materials and methods

Ethics statement

This retrospective pilot study is compliant with the Health Insurance and Accountability Act of 1996 (HIPA-A) and was approved by the Institutional Review Board of William P. Clements Jr. University Hospital, Dallas, Texas (STU-102012-084). Written informed consent was obtained for patients prior to treatment.

Patients

This retrospective, single-center pilot study included a total of 35 patients with HCC who underwent Y90 TARE. The study included adult patients, aged 18 or above, who were diagnosed with HCC between January 2020 and December 2021. Only patients without evidence of extrahepatic metastases and who underwent Y90 therapy after discussion in a multidisciplinary tumor board were included. Patients who had recent esophageal or gastric bleeding, uncontrolled hepatic encephalopathy, cardiac failure, renal failure, or sepsis were excluded from the study. Seventy-seven percent (n = 22) of patients were male. In terms of race/ethnicity, 63% were White non-Hispanic, 17% were Black non-Hispanic, and 21% were identified as Hispanic.

HCC diagnosis was based on characteristic imaging using the Liver Imaging Reporting and Data System version 2018 (LI-RADS, American College of Radiology, USA) [14]. After Y90 therapy, patients were followed up with contrast-enhanced CT or MRI scans at three-month intervals. This follow-up continued until the multidisciplinary board recommended a change in therapy.

Y90 TARE procedure

Patients were treated with TARE using Y90 glass microspheres (TheraSphere, Boston Scientific, USA). All patients underwent prior mapping to establish the intended plan of treatment, dosimetry, and calculate the lung shunt fraction using technetium-99m macroaggregated albumin (Tc-99m MAA) and single-photon emission computed tomography-computed tomography (SPECT-CT). Target radiation dose was calculated based on single-compartment Medical Internal Radiation Dose (MIRD) dosimetry. The intent of treatment was defined as curative using radiation segmentectomy and lobectomy when indicated. Angiosomes were calculated based on the segmentation using cone beam CT (CBCT) at the time of the mapping. Palliative treatments were administered to patients with multifocal unilobar or bilobar disease with or without portal vein invasion to delay or prevent progression and to downstage and possibly convert to curative therapy.

Data collection

Clinical data included patient age, sex, race/ethnicity, etiology of cirrhosis, and Eastern Cooperative Oncology Group (ECOG) performance status. Tumor data of interest included number of HCC nodules treated, maximum diameter, and Barcelona Clinic Liver Cancer (BCLC) stage. We also collected pathology findings, either by biopsy or explant, when available. Laboratory values taken one to seven days prior to treatment included white blood cells (WBCs), platelet count (P), neutrophils (N), lymphocytes (L), sodium, alkaline phosphatase (AP), aspartate aminotransferase (AST), alanine aminotransferase (ALT), albumin, total bilirubin, and alpha-fetoprotein (AFP).

Liver fibrosis, liver function biomarkers, and systemic inflammation indexes were calculated with these formulas: 1) APRI index = AST patient level/AST (upper limit of normal) / platelet count (109/L) x 100 [15]. 2) FIB-4 = age (years) x AST (U/L)/platelet count (109/L) x [16]. 3) ALBI score = 0.085 x (albumin/L) + 0.66 lg (total bilirubin µmol/L) [7]. 4) MELD score = 9.6 loge (creatinine mg/dl) +3.8 loge (bilirubin mg/dl) +11.2 loge (INR) + 6.4 [17]. 5) MELD sodium = MELD + 1.32 × (137 − Na) − (0.033 × MELD × [137 − Na]) [18]. 6) SII = P x N /L x 109 cells/L [19]. 7) NLR = N/ L [19]. 8) PLR = P/L. [19].

Tumor response assessment

Radiologic tumor response was assessed on a per-patient basis using the modified Response Evaluation Criteria in Solid Tumors (mRECIST) for HCC [20]. Patients were evaluated every three months after starting TARE. Depending on the disease progression and medical advice, some patients received one, two, or three treatments. The assessment response was evaluated in the third, sixth, and ninth months, considering the response observed during the last assessment. Tumor response was categorized into two levels; patients with complete response (CR) or partial response (PR) were classified as “responders," and patients with stable disease (SD) or progressive disease (PD) were classified as “non-responders”.

Statistical analysis

Descriptive statistics were used to analyze the clinical and laboratory data collected. Continuous variables were expressed as mean (and standard deviation: SD) or median (range), and categorical variables were expressed as counts and percentage of patients. T-test and the Wilcoxon rank-sum test were used to compare continuous variables, while the chi-square was used to compare categorical variables.

Classification and regression tree (CART) analysis was used to calculate the cutoff values for each biomarker [21]. Each biomarker was dichotomized according to the cut-off value. Sensitivity, specificity, area under the curve (AUC), and positive and negative likelihood ratios were calculated for each biomarker to determine the best predictor in each category. The positive likelihood ratios (+LR) for the cutoff values ranged from ~1 to 10. An LR close to 1 indicated that the test result did not change the likelihood of the outcome of interest, while values of 1-2 for the positive likelihood ratio (+LR) indicate a minimal increase in the probability of the outcome, values greater than 2 to 5 indicate a small increase, and values greater than 5 to 10 indicate a moderate or high probability. The negative likelihood ratio (-LR) ranges from 1 to 0. A value of 1 does not change the probability of the outcome, values greater than 0.5 to 1.0 indicate a minimal decrease in the likelihood of the outcome, values greater than 0.2 to 0.5 indicate a small decrease, values from 0.1 to 0.2 indicate a moderate decrease, and values less than 0.1 indicate a large decrease in the likelihood of disease. Correlation analysis was utilized to determine the association between continuous variables, which was assessed using Pearson’s correlation coefficient (rho = r). This analysis facilitated the selection of biomarkers for inclusion in the multivariable model while avoiding multicollinearity.

Logistic regression was employed to establish the association between predictors or biomarkers and tumor response. Due to the constraints of the sample size, demographic and clinical variables with three or more categories were dichotomized into two levels for the bivariate logistic regression analysis, thereby enhancing the statistical power. In the multivariable logistic regression analysis, predictors, and biomarkers with a p-value of less than 0.2 were selected to evaluate their association with tumor response. Biomarkers demonstrating a correlation greater than 0.3 were excluded from the analysis to prevent multicollinearity. The multivariable logistic regression model performance was tested using accuracy, sensitivity, precision, F1 score, and AUC. F1 score minimizes false positives and false negatives. Statistical analyses were conducted using Minitab® (Minitab, LLC, USA) and SAS OnDemand for Academics® (SAS Institute, USA), and a two-sided p-value of less than 0.05 was considered statistically significant for all analyses. Graphic visualization was used with Python 9.3 (Python Software Foundation, USA).

## Results

Patient characteristics

Table 1 presents an overview of key findings from patients who underwent Y90 treatment. The average age of participants was 66.4 years (SD: 10), and the average BMI was 28.5 (SD: 8.8). Among the participants, 89% (31/35) had cirrhosis. The leading causes of cirrhosis were viral factors (37%, 13/35), and non-alcoholic steatohepatitis (NASH) (37%, 13/35). Hepatic nodules were found in varying numbers and sizes: 41% (14/35) had one nodule, 32% (11/35) had two to three nodules, 27% (9/35) had four or more nodules, and 59% (19/35) of the patients had nodules with a diameter between 2 and 5 cm. The predominant BCLC classification was stage B (46%, 16/35), followed by stage C (26%, 16/35). LI-RADS classifications were predominantly LR-5 (89%, 31/35). The number of Y90 treatments varied: 63% (22/35) of patients received three treatments, 34% (12/35) received two treatments, and 3% (1/35) received one. Types of Y90 treatment included radiation segmentectomy (63%, 22/35), lobectomy (23%, 8/35), and standard TARE (15%, 5/35). The mean Y90 doses for each treatment can be consulted in Table 1. mRECIST response assessment indicated CR in 14%, PR in 37%, SD in 46%, and PD in 3% of patients. Overall response assessment showed a positive or good response in 51% and non-response in 49% of patients. For complete details, refer to Table 1. The mean follow-up after the last Y90 treatment was 7.8 months.

**Table 1:** Demographic characteristics of patients with HCC submitted to Y90 (n = 35). Abbreviations: NH: non-Hispanic, NASH: non-alcoholic steatohepatitis, HCV: hepatitis C virus, HBV: hepatitis B virus, Vol: volume, ECOG: Eastern Cooperative Oncology Group, cm: centimeters, BCLC: Barcelona Clinic Liver Cancer, LT: liver transplant, LIRADS: Liver Imaging Reporting and Data System, LR: LIRADS, TIV: tumor in vein, LRTR: LIRADS treatment response, E: equivocal, N: non-viable, V: viable, seg: segmentectomy, mRECIST: modified RECIST, CR: complete response, PR: partial response, SD: stable disease, PD: progressive disease

Variable	Number	%
Age, mean, sd.	66.4	10
BMI, mean, sd.	28.5	8.8
Sex		
Female	8	23%
Male	27	77%
Ethnicity		
Whites NH	22	63%
Black NH	6	17%
Hispanic + others	7	21%
Cirrhosis		
No	4	11%
Yes	31	89%
Etiology		
Viral ECV/EBV	13	37%
Ethanol	4	11%
NASH	13	37%
No etiology	5	14%
Ascites		
No	26	74%
Yes	9	26%
Large-volume paracentesis		
No	30	86%
Yes	5	15%
ECOG status		
ECOG 1	31	89%
ECOG 2	4	11%
Number of hepatic nodules		
One nodule	14	41%
Two to three nodules	11	32%
Four or more nodules	9	27%
Diameter of nodules (cm)		
Below 2 cm	4	12%
Between 2 and 5 cm	19	59%
More than 5 cm	12	34%
Portal vein thrombosis		
No	30	86%
Yes	5	14%
BCLC		
Stage 0	2	6%
Stage A	8	23%
Stage B	16	46%
Stage C	9	26%
LIRADS		
LR-4	2	6%
LR-5	31	89%
LR-TIV	2	6%
LIRADS post-treatment		
LRTR-E	22	63%
LRTR-N	4	11%
LRTR-V	9	26%
Intention of treatment		
Bridging therapy	9	26%
Downstaging	19	56%
Palliative treatment	6	18%
Number of Y90 treatments		
One treatment	1	3%
Two treatments	12	34%
Three treatments	22	63%
Type of Y90 treatment		
Radiation seg	22	63%
Lobectomy	8	23%
Standard T	5	15%
Mean Y90 dosage, mean (SD)		
Radiation segmentectomy, mean (SD)	361 Gy	175 Gy
Lobectomy, mean (SD)	134 GY	8 Gy
Standard T, mean (SD)	153 Gy	57 Gy
Pathology explanted liver		
Non-viable (total necrosis)	6	17%
Viable (partial necrosis)	3	9%
mRECIST		
CR	5	14%
PR	13	37%
SD	16	46%
PD	1	3%
Response assessment		
Response	18	51%
Non-response	17	49%

Main characteristics between responders and non-responders and predictive cutoff values

The mean value of albumin was higher among responders than non-responders (4.1 vs. 3.6, p < 0.02. A similar trend was observed for the mean ALBI score; however, this did not reach statistical significance (-2.8 vs. -2.4, p = 0.07). On the other side, the MELD sodium was higher in responders than non-responders (11 vs. 9, p = 0.040, respectively) (see Table 2).

**Table 2:** Biomarker distribution in responders and non-responders. Abbreviations: * Non-parametric t-test, SD: standard deviation, BMI: body mass index, ALT: alanine aminotransferase, AST: aspartate aminotransferase, P: phosphatase, INR: international normalized ratio, AFP: alpha-fetoprotein, FIB-4: liver fibrosis assessment based on four factors, APRI: aspartate aminotransferase to-platelet ratio index, ALBI: albumin bilirubin, MELD: model for end-stage liver disease, SII: systemic immune index, NLR: neutrophil-to-lymphocyte ratio, PLR: platelet-to-lymphocyte ratio

	Overall sample		Responders		Non-responders		
Variable	Mean	SD	Mean	SD	Mean	SD	P-value
Sodium	138	2.7	138	2.9	139	2.6	0.7
Creatinine	1	0.3	1	0.3	1	0.3	0.8
Albumin	3.9	0.6	4.1	0.5	3.6	0.6	0.02
ALT	60.9	63.4	70.8	84	50.4	29.4	0.8*
AST	68.2	56	73	69	63.2	40.6	0.7*
Alkaline p.	118.7	46.7	118.3	40.3	119	54	0.7*
Bilirubin	1	1.2	1.2	1.7	0.7	0.3	0.9
INR	1.1	0.2	1.1	0.2	1	0.1	0.3
WBC	4.8	2.7	5.1	2.9	4.5	2.4	0.5*
Lymphocytes	1.1	0.4	1.1	0.4	1	0.5	0.8*
Neutrophils	3.1	2.2	3.2	2.6	3.1	1.7	0.7*
Platelets	135.4	87.8	125.4	71.6	146	103	0.7*
AFP	514	1207	396	886	639	1494	0.9*
FIB-4 index	6.8	5.7	7.5	6.9	6.1	4.1	0.8*
APRI index	2.2	3.4	2.8	4.7	1.5	1.1	0.8*
ALBI score	-2.6	0.6	-2.8	0.6	-2.4	0.5	0.07
MELD	8	3	9	3	8	2	0.1
MELD sodium	10	3	11	3	9	2	0.04
SII	468.4	485.5	433.6	516.7	505.1	463	0.5*
NLR	3.2	1.8	3.1	1.8	3.4	1.8	0.3*
PLR	131.4	73.7	116.3	51.4	147	90.5	0.4*

Table 3 describes the cutoff values of the biomarkers, which can be used to separate responders from non-responders. The cutoff value of the ALBI score and NLR had larger +LR (3.1 and 3.2) and smaller -LR (0.55 and 0.7) compared with other response predictors. The sample size was insufficient to calculate the +LR and -LR for the cutoff values of FIB-4, SII, and PLR.

**Table 3:** Biomarkers' cut-off values, sensitivity, specificity, +likelihood, -likelihood ratio, odds ratios, and area under the curve. Abbreviations: +LR: positive likelihood ratio, -LR: negative likelihood ratio, OR: odds ratio, AUC: area under the curve, FIB-4: liver fibrosis assessment based on four factors, APRI: aspartate aminotransferase-to-platelet ratio index, ALBI: albumin bilirubin, MELD: model for end-stage liver disease, SII: systemic immune index, NLR: neutrophil-to-lymphocyte ratio, PLR: platelet to lymphocyte ratio, NA: not applicable

Biomarker	Cutoff value	Sensitivity	Specificity	+LR	-LR	OR	AUC	P-value
Albumin	<=3.8	0.64	0.72	2.29	0.5	4.7	0.69	0.03
FIB-4 Index	<=14.4	0.72	0	NA	NA	0	0.63	0.9
APRI index	<=0.3435	0.16	0.94	2.7	0.89	3.2	0.55	0.3
ALBI score	<=-2.885	0.55	0.82	3.1	0.55	5.8	0.69	0.02
MELD	<=10	0.66	0.11	0.7	3.1	0.3	0.6	0.14
MELD sodium	<=9	0.33	0.23	0.43	2.9	0.15	0.71	0.01
SII	<=115.6	0.16	1	NA	NA	0	0.58	0.9
NLR	<=1.92	0.38	0.88	3.2	0.7	4.7	0.63	0.08
PLR	<=229.4	1	0.17	NA	NA	0	0.58	0.96

Correlation analysis

Correlation analysis revealed that FIB-4 was correlated with APRI (rho(r) = 0.7, p = 0.0001) and ALBI score (r = 0.4, p = 0.04). MELD showed a significant correlation with MELD sodium (r = 0.9, p < 0.001) and ALBI score (r = 0.4, p < 0.004). Systemic inflammation indexes, such as SII, NLR, and PLR, exhibited significant correlations with each other (r > 0.7, p < 0.001). Moreover, MELD sodium demonstrated a moderate correlation with the ALBI score (r = 0.35, p = 0.004), thereby necessitating caution when combining these variables in the multivariable logistic regression analysis to avoid potential confounding effects. No correlation was observed between the ALBI score and systemic immune-inflammatory markers (see Table 4).

**Table 4:** Matrix of correlation analysis among biomarkers and coefficient correlation (rho= r, and p values). Abbreviations: r: rho coefficient, p: p-value, FIB-4: liver fibrosis assessment based on four factors, APRI: aspartate aminotransferase-to-platelet ratio index, ALBI: albumin bilirubin, MELD: model for end-stage liver disease, SII: systemic immune index, NLR: neutrophil to lymphocyte ratio, PLR: platelet-to-lymphocyte ratio

Biomarker	FIB-4	APRI	ALBI	MELD	MELDna	SII	NLR	PLR
FIB-4	1							
APRI. r (p)	0.7 (.0001)	1						
ALBI r (p)	0.4 (0.04)	0.08 (0.6)	1					
MELD r (p)	0.2 (0.27)	0.2 (0.25)	0.4 (0.02)	1				
MELDna r (p)	0.12 (0.5)	0.16 (0.4)	0.35 (0.004)	0.9 (0.0001)	1			
SII r (p)	0.5 (0.002)	0.3 (0.07)	0.07 (0.7)	-0.09 (0.5)	-0.03 (0.9)	1		
NLR r (p)	-0.02 (0.3)	-0.18 (0.3)	0.2 (0.2)	0.07 (0.7)	0.1 (0.6)	0.8 (0.0001)	1	
PLR r (p)	-0.45 (0.007)	-0.33 (0.05)	0.08 (0.6)	-0.13 (0.4)	-0.14 (0.4)	0.7 (0.0001)	0.64 (0.0001)	1

Logistic regression analysis and model performance

In the bivariate logistic regression analysis, the demographic and clinical predictors showed no association with the tumor response (Table 5). In addition, predictors related to Y90 therapy were not included in this analysis due to limitations imposed by the sample size. On the other hand, the data revealed that lower ALBI scores and lower NLR values were positively associated with a favorable tumor response. Specifically, patients with ALBI scores of ≤-2.8 had a significantly higher probability of a good response, with an odds ratio (OR) of 5.8 (95% CI: 1.2-27.6, p = 0.03). Similarly, patients with an NLR of ≤1.92 were more likely to have a good response, although this association did not reach statistical significance (OR: 4.7, 95% CI: 0.8-27.5, p = 0.08). On the contrary, a lower MELD sodium value (<9) was associated with a reduced likelihood of a good response, as indicated by an OR of 0.15 (95% CI: 0.04-0.7, p = 0.014), translating to an 85% decrease in the probability of achieving a positive outcome after Y90 treatment. To interpret the ORs for ALBI scores and NLR, patients with ALBI scores lower than -2.8 had 5.8 times more probability of a good response than those with values greater than -2.8. Similarly, patients with an NLR of ≤1.92 had 4.7 times more probability of a good response compared to those with values above 1.92; however, the association for NLR was not statistically significant.

**Table 5:** Logistic regression analysis and association between predictors and tumor response (n = 35). Abbreviations: OR: odds ratio, 95%CI; confidence intervals, APRI: aspartate aminotransferase-to-platelet ratio index, ALBI: albumin bilirubin, NLR: neutrophil-to-lymphocyte ratio, MELD: model for end-stage liver disease

Variable	OR	95%CI	P-value
Age	0.979	0.91-1.05	0.5527
BMI	0.888	0.76-1.04	0.1453
Sex			
Female	0.929	0.19-4.5	0.9266
Male	Reference		
Ethnicity			
Whites NH	Reference		
Other ethnicities	0.857	0.22-3.39	0.8261
Cirrhosis etiology			
Viral	Reference		
Other etiologies	0.711	0.19-2.69	0.6156
Ascites			
No	1.458	0.32-6.7	0.6276
Yes	Reference		
Number of hepatic nodules			
One nodule	0.716	0.19-2.74	0.6259
Two or more nodules	Reference		
Diameter of nodules			
Below 5 cm	1.091	0.27-4.41	0.9028
Equal or more than 5 cm	Reference		
Number of Y90 treatments			
One to two treatments	0.714	0.18-2.83	0.6317
Three treatments	Reference		
APRI index			
<=0.3435	3.2	0.3-34	0.33
>0.3435	Reference		
ALBI score			
<=-2.885	5.8	1.2-27.6	0.03
>-2.885	Reference		
NLR			
<=1.92	4.7	0.8-27.5	0.08
>=1.92	Reference		
MEDL sodium			
<=9	0.15	0.04-0.7	0.014
>9	Reference		

A multivariable regression analysis was executed, encompassing variables exhibiting a correlation of less than r = 0.2 (Table 4) and those with p < 0.2 from the bivariate analysis (as shown in Table 5). The MELD score was excluded given its low +LR and correlation with the ALBI score. In the multivariable model, tumor response retained an independent association with ALBI scores of ≤-2.8 (OR 6.1, 95% CI 2.7-14.4, p = 0.03). However, although the magnitude of the association (OR) between the cutoff value of NLR and tumor response was significant (greater than 2), its p-value did not attain statistical significance (OR 5.1, 95% CI 0.8-11.9, p = 0.08) (Table 6).

**Table 6:** Association between biomarkers and tumor response in the multivariable logistic model. Abbreviations: AOR: adjusted odds ratio, 95%CI; confidence intervals, ALBI: albumin bilirubin, NLR: neutrophil-to-lymphocyte ratio

Biomarker	AOR	95%CI	P value
ALBI score			
<=-2.885	6.1	2.7-14.4	0.03
>-2.885	Reference		
NLR			
<=1.92	5.1	2.2-11.9	0.08
>=1.92	Reference		

**Figure 1:**
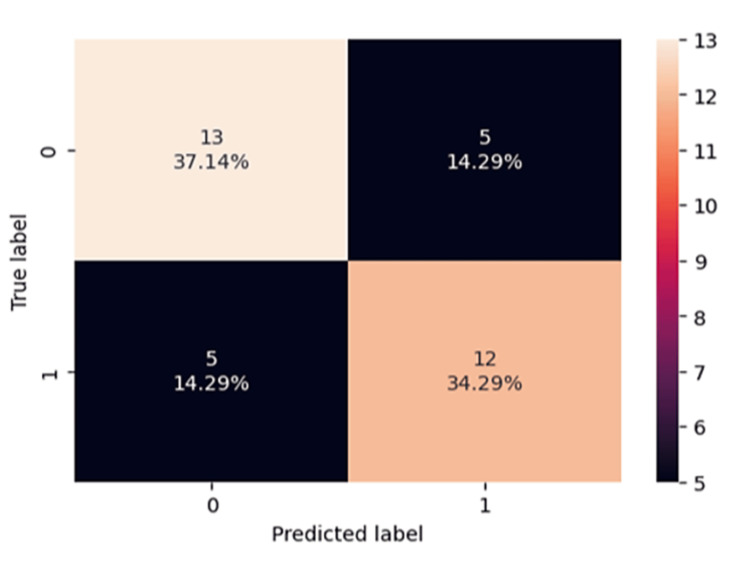
Confusion matrix and performance for the multivariable logistic regression model. Confusion matrix and performance for the multivariable logistic regression model including the ALBI and NLR groups. True label: 0 = responders, 1 = no responders. Predicted label: 0 = responders, 1 = no responders.

**Figure 2:**
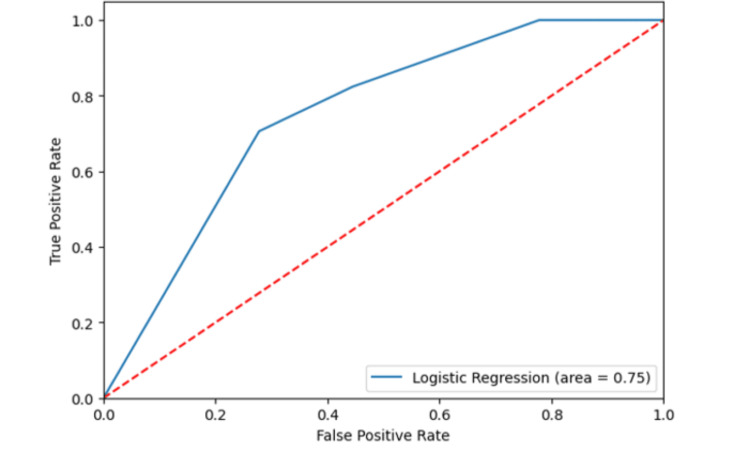
The area under the curve of the multivariable model with ALBI and NLR cutoff values and tumor response. ALBI: albumin-bilirubin, NLR: neutrophil-to-lymphocyte ratio

The performance of the multivariable model showed an accuracy of 0.71, a sensitivity of 0.71, a precision of 0.71, and an F1 of 0.71. The area under the curve (AUC) for the multivariable model, used for predicting treatment response, was 0.75 (see Figures 1, 2).

In the simulated logistic regression analysis, increasing the sample size twofold, the association between treatment response and NLR could become statistically significant (p = 0.02).

## Discussion

The findings of this research suggest that the ALBI score is a significant predictor of tumor response following Y90 therapy, as evidenced by its statistical significance in the multivariate analysis. While the NLR displayed a promising OR, its p-value did not reach the conventional threshold for statistical significance. This marginal significance may be attributed to the limited sample size of the study. Furthermore, both biomarkers can be used in combination. Future research should involve a larger cohort to enhance the statistical power and potentially confirm the predictive value of NLR in conjunction with the ALBI score for tumor response post-Y90 therapy.

Studies in cancer have demonstrated that bilirubin suppresses CD4+ T cell responses, impacting antitumor immunity. Albumin, reflecting nutritional status and inflammation, influences patient survival [22]. The NLR mirrors the tumor immune microenvironment. Research suggests that a particular neutrophil subpopulation may downregulate CD4+ T cell proliferation and activity [22]. These biomarkers hold promise for predicting outcomes and guiding HCC management.

The aim of this study was to investigate the potential impact of combining pretreatment biomarkers associated with liver fibrosis, liver function, immune inflammation, and tumor response following TARE.

Previous research has demonstrated that changes in the ECM can affect the exchange of substances between the blood and hepatocytes [23]. In addition, the degree of fibrosis has been linked to the tumor diameter, with increased fibrosis correlating with smaller tumor sizes and the presence of a complete capsule in the liver [24]. Furthermore, Mai et al. [25] found that the combination of ALBI and APRI could be used to predict post-hepatectomy liver failure (PHLF) in hepatitis B virus-related HCC. Luo et al. [6] showed that the combination of ALBI grade and APRI score with a cutoff value of <0.5 may be used to predict OS in patients with HCC within the Milan criteria following liver resection. The findings of the current study did not show a relationship between liver fibrosis biomarkers, such as the APRI index and FIB-4, and tumor response to Y90 therapy. Simulated analysis with a larger sample size did suggest a statistically significant association with the APRI index; however, further investigation needs to be conducted to validate this result with a larger subject population.

Patients with HCC who have a high pre-treatment ALBI grade (grades 2 and 3 or scores >-2.6) tend to experience a poorer prognosis when undergoing TACE therapy. Extensive research has investigated the prognostic value of ALBI grade, consistently identifying it as a predictor for overall prognosis, including OS and recurrence-free survival, in patients receiving liver resection or liver transplantation [26]. ALBI grade has also been found to be a prognostic factor after a variety of locally directed liver therapies, such as microwave ablation, radiofrequency ablation, TACE, and TARE [27, 28]. Specifically, for TARE, a study found that ALBI grade was a stronger prognostic indicator than C-P class in predicting OS among HCC patients treated with TARE, particularly in the subgroup of C-P class A patients. Patients with ALBI grade 1 had a superior OS compared to those with ALBI grade 2. The study also identified alanine transaminase, Barcelona Clinic Liver Cancer stage, and ALBI grade as the strongest prognostic factors for OS. These findings suggest that ALBI grade is a useful tool for clinicians to make decisions on recommending TARE treatment to patients with HCC [29]. Furthermore, other studies have demonstrated the prognostic value of combining ALBI with other biomarkers, such as APRI, FIB-4, and NLR [6,25,27]. Soydal et al. in a multivariable analysis found that in patients receiving TARE for unresectable HCC, INR, alfa-fetoprotein, ALBI grade, NLR, Child-Pugh score, and ascites were the main predictors of OS [27].

Previous studies have also demonstrated that elevated NLR is linked to worse OS, disease-free survival, and recurrence-free survival after hepatic resection, curative surgical resection of HCC, LT, locally directed therapies, intraarterial therapies, and palliative systemic therapies [12,13,29].

Schobert et al. found that low NLR and PLR were correlated with well-defined tumor or high tumor sphericity as assessed with radiomics in MRI. In addition, low NLR and PLR values were associated with good outcomes in terms of objective response to treatment [30].

There is a paucity of literature utilizing NLR in conjunction with other biomarkers, such as demonstrated in the Soydal study. The authors of this study found that in unresectable HCC, both ALBI grade and NLR greater than 5 were associated with poorer OS in HCC patients receiving TARE [27].

This study, along with the previous one, successfully demonstrated a connection between the ALBI score and objective tumor response in patients with HCC. The study also suggested a potential synergistic relationship between the ALBI score and NLR in predicting HCC TARE response. This insight provides a novel approach to assessing treatment outcomes by combining two readily accessible markers.

There are several limitations in this study, with the most significant being the small sample size. This limitation may have hindered the ability to detect more subtle associations and could compromise the generalizability of the findings to a broader population of HCC patients. In addition, due to the small sample size, the study was unable to establish cutoff values for FIB-4 and SII indexes. The impact of the small sample size on statistical power was evident, particularly in relation to NLR and APRI indexes. Furthermore, the small sample size prevents the establishment of associations between predictors with three or more categories. Further research is necessary to determine appropriate cutoff values for predictors that consider the outcome of the intervention.

## Conclusions

The ALBI score is a reliable predictor of treatment response following TARE. The NLR index may offer further prognostic information, and both biomarkers can be used in combination. However, further research involving a larger sample size is necessary to confirm the NLR's statistical significance.
